# Use of Natural and Synthetic Fiber-Reinforced Composites for Punching Shear of Flat Slabs: A Comparative Study

**DOI:** 10.3390/polym14040719

**Published:** 2022-02-13

**Authors:** Panuwat Joyklad, Ekkachai Yooprasertchai, Pongsak Wiwatrojanagul, Krisada Chaiyasarn, Nazam Ali, Qudeer Hussain

**Affiliations:** 1 Department of Civil and Environmental Engineering, Faculty of Engineering, Srinakharinwirot University, Nakhonnayok 26120, Thailand; panuwatj@g.swu.ac.th; 2 Construction Innovations and Future Infrastructure Research Center (CIFIR), Department of Civil Engineering, Faculty of Engineering, King Mongkut’s University of Technology Thonburi, Bangkok 10140, Thailand; 3 Theta Forge, Ladprao, Bangkok 10230, Thailand; pongsak.wiwat@outlook.com; 4 Thammasat Research Unit in Infrastructure Inspection and Monitoring, Repair and Strengthening (IIMRS), Thammasat School of Engineering, Faculty of Engineering, Thammasat University Rangsit, Rangsit 12121, Thailand; ckrisada@engr.tu.ac.th; 5 Department of Civil Engineering, School of Engineering, University of Management and Technology, Lahore 54770, Pakistan; nazam.ali@umt.edu.pk; 6 Center of Excellence in Earthquake Engineering and Vibration, Department of Civil Engineering, Chulalongkorn University, Bangkok 10330, Thailand; ebbadat@hotmail.com

**Keywords:** flat slabs, natural fibers, synthetic fibers, punching shear, fiber-reinforced polymers, concrete

## Abstract

Over the last two decades, considerable attention has been devoted to the strengthening of sub-standard flat-slab constructions. With the evolution of composite materials and an increasing emphasis on the economical and sustainable use of natural fibers, many researchers have utilized them in the strengthening of flat flabs mitigating punching failures. This study aims at investigating and comparing the behavior of flat slabs strengthened with post-installed composite and natural reinforcements. An experimental program was devised consisting of eight flat-slab specimens. One specimen was tested in as-built condition to provide a reference. The remaining specimens were strengthened with Carbon Fiber-Reinforced Polymer (CFRP), Aramid Fiber-Reinforced Polymer (AFRP), and sisal rods. The pattern of post-installed rods was varied as single line, double line, and star shapes around the column. The results indicated that the single-line pattern could only enhance the maximum sustained load by up to 6% compared to that of the reference specimen. On the contrary, double line and star shape configurations resulted in a substantial increase in the maximum sustained load. An analytical assessment of ACI 318-19 provisions resulted in an over-estimation of the shear strengths of CFRP- and AFRP-strengthened slabs. Furthermore, the same provisions led to lower yields than experimental shear strengths for sisal-strengthened slabs.

## 1. Introduction

Reinforced concrete (RC) flat plate slabs are kind of structural components that find their supports directly on columns. Relative to conventional RC slab-beam structures, they involve easier and cheaper constructions, lesser complications, and lower story heights [1]. The direct connection of flat slabs on columns means that these are vulnerable to punching shear as a result of the high shear stresses that accumulated in the neighborhood of connections [2,3]. In the event when the induced shear stress exceeds the shear capacity of flat slabs around columns, inclined cracks appear, which eventually lead to their punching around columns [4]. Failures associated with punching shear are worth contemplating due to their instantaneous and brittle nature [5,6,7]. These failures are associated with negligible post-failure rotational capacity and ductility, which could result in a progressive collapse of the structure [1].

One way to mitigate punching failure is by furnishing flat slabs with sufficient shear reinforcement, complying with current design requirements. However, many of the existing flat slabs do not incorporate adequate shear reinforcement, imperiling them to possible punching failures. The strengthening of such deficient flat slabs has been investigated by researchers. This mainly involves the installation of steel bolts or other shear reinforcement in the vicinity of slab–column connections. Post-installation of drop panels or column capitals can theoretically enlarge shear-resisting areas around columns. However, for practical reasons, including architectural and aesthetic issues, these methods are seldom practiced [8,9]. Many studies have proved the effectiveness of post-installed steel bolts to enhance the punching capacities of flat slabs. Baig and Abbas [10] tested four full-scale flat-slab representatives of interior beam column connections. Two specimens were constructed with a concrete strength of 14 MPa, while the remaining two were constructed with a concrete strength of 28 MPa. One specimen was tested for each concrete strength in as-built condition, while the other specimen was strengthened with post-installed steel bolts. It was concluded that the application of post-installed steel bolts successfully altered the failure mode from brittle shear (as found in control specimens) to a ductile one. Furthermore, ultimate load capacities were enhanced by approximately 64–73%. Bu and Polak [11] retrofitted slab-column connections with shear bolts tested under cyclic lateral drift and constant gravity loads. It was reported that shear-bolt-strengthened specimens were able to withstand higher ultimate loads and corresponding deflections, exhibited a more ductile response, and dissipated higher energies in comparison to their counterpart control specimens. El-Salakawy et al. [12] proposed a new technique for strengthening deficient flat slabs using post-installed shear bolts. The new strengthening technique comprised the external installation of shear bolts in holes drilled through the slab thickness. The shear bolt comprised a headed vertical rod, threaded at the other end for fixing using the washer-nut method. A total of six slabs were tested, each measuring 1540×1020×120 mm with concentric square columns of the size 250×250 mm. Four slabs were strengthened with shear bolts, while the remaining two were tested in as-built condition. It was reported that strengthened slabs experienced a substantial increase in punching capacity and ductility. Other researchers [13,14,15] have also reported the positive effects of steel bolts to mitigate punching failures. Although steel bolts have proved their effectiveness in minimizing the dangers associated with punching failures, their vulnerability to corrosion has been a concern [1].

Alternative methods have been investigated by researchers, which primarily revolve around the application of Fiber-Reinforced Composites (FRPs). This could be attributed to their distinctive characteristics, including a high tensile strength, resistance to corrosion, low weight, and simple application procedures [16,17,18,19]. Meisami et al. [20] tested five flat-slab-column connections with one slab in as-built condition and the remaining four slabs strengthened with Carbon Fiber-Reinforced Polymers (CFRP) grids. CFRP grid configurations were varied for three specimens as 8, 16, and 24 post-installed grids. One specimen was furnished with a pre-installed CFRP grid. Using the adopted strengthening technique, brittle shear failure, as found in the control specimen, was successfully altered to a ductile one. The dominant failure mode in strengthened specimens was observed to be the debonding of CFRP. Erdogan et al. [21] tested seven representative specimens of interior slab-column connections. Specimens were strengthened with in-house fabricated CFRP dowels in different numbers and configurations. The test results indicated that the ultimate capacities of strengthened specimens improved by up to 1.3 times compared to that of the control specimen. Binici and Byrak [22] presented a technique to increase the punching capacity of flat plate slabs. They vertically employed CFRP strips within the vicinity of the column’s concentrated load. It was reported that the punching capacities of flat plate slabs increased with the increased number of CFRP strips. Relative to control specimen, punching capacities were increased by up to 51%.

Yooprasertchai et al. [23] tested seven representative specimens of typical deficient flat-slabs. One slab was tested in as-built condition, whereas the other six specimens were strengthened with GFRP rods. GFRP rod configurations were varied, including single- and double-cross patterns as well as radial patterns. It was found that radial configuration resulted in the greatest improvement in ultimate capacities and corresponding deformation capacities. Furthermore, a reduction in GFRP rods’ spacing increased the magnitudes of peak sustained loads. However, this increase was not found to be proportional with the reduction in GFRP rod spacing. Yooprasertchai et al. [24] investigated the performance of CFRP and aramid FRP (AFRP) in enhancing the punching capacities of flat slabs relative to conventional steel bolts. Shear reinforcement was varied in two ratios, comprising four and seven bars on each column’s side, equivalent to 0.75- and 0.5-times the slab’s effective depth, respectively. All shear reinforcements were arranged in a single line cruciform pattern. The results concluded that both CFRP and AFRP could effectively enhance load-capacity and ductility. Other studies [25,26,27,28] have also highlighted the effectiveness of FRP composites in altering the brittle punching shear failure to flexural failure. Recently, the potential use of natural fibers as an alternative to synthetic FRP in the strengthening of RC structures has been explored. De Azevedo et al. [29] suggested that natural fibers exhibited promising potential in augmenting the flexural strength of geopolymer matrices. Several researchers [30,31,32,33,34,35,36] highlighted the ability of natural fibers in improving the structural behavior of RC beams. Others [37,38] used natural fibers in the renovation of RC columns. Among the various natural fibers that are available, Sisal has been shown to demonstrate excellent strengthening attributes for RC structures. Khan et al. [39] strengthened RC beams using sisal fiber sheets and rods. They concluded that both sisal sheets and rods augmented to the ultimate load capacities of strengthened beams. Okeola et al. [40] concluded that the sisal fiber-reinforced concrete exterior beam-column joints tested under monotonic loading demonstrated a higher yield and ultimate load, lower deflections, and higher shear strength. Sharda et al. [41] studied the axial compression of the all-composite modular wall system. The study demonstrated the high potential of fiber composite materials. Al-Fakher et al. [42] investigated the bending behavior of concrete slabs by using externally attached FRP tubes. The FRP tubes improved the structural performance.

At present, different types of rods (such as steel, synthetic and natural FRP) are available, which can be utilized to enhance the punching shear of the flat slabs and structural performance of concrete members [43,44,45,46]. However, their efficiency in enhancing the punching shear of flat slab is rare [23,24] especially where there is concern about the comparative performance. The conventional steel bolts are frequently available all around the world; however, there are some issues, such as corrosion and weight. On the other hand, both the CFRP and AFRP are considered high-performance, lightweight and non-corrosive. There are some concerns with the use of synthetic FRP rods, such as their relatively high cost and environmental impacts. Regarding sustainability and the green environment, FRP rods could be much more useful. Therefore, natural sisal FRP rods were also chosen, owing to their sustainability and low cost, and natural resource. A detailed review of existing studies indicates that, to date, a direct comparison of different types of rods has not been published. There is need to investigate the comparative performance of different FRP rods to establish further design guidelines and recommendations. Therefore, this study focusses on comparative experimental investigations between CFRP, AFRP, conventional steel bolts, and Sisal in improving the behavior of deficient flat slabs. Additionally, no studies have been published using different types of rods and configurations of shear reinforced in flat slabs for strengthening purposes. Further, different configurations of aforesaid shear reinforcements are studied, to look into the optimum combination of the type of shear reinforcement and its configuration within the vicinity of columns in flat slabs.

## 2. Materials and Methods

### 2.1. Test Matrix

The test matrix comprised 8 flat-slab specimens. One specimen was tested in as-built condition, whereas the remaining 7 specimens were strengthened using either post-installed FRP composites or sisal rods. A detailed description of the test matrix is presented in Table 1. Specimens were strengthened using CFRP, sisal, or Aramid FRP (AFRP) rods around the columns. Rods were either in a cross or star form, as shown in Table 1. Table 1 presents a detailed description of the 8 specimens tested in this study. Specimen 1-CTRL was tested in as-built condition. Specimens 2-CP-1RC and 3-CP-1RS were strengthened using CFRP rods in single layers of cross and star patterns, respectively. Specimens 4-SL-2RC and 5-SL-2RS were strengthened with 2 layers of sisal rods in cross and star patterns, respectively. Specimens 6-CP-2SC and 7-CP-2SS were strengthened with CFRP sheet rods in 2 layers of cross and star patterns, respectively. The last specimen 8-AP-1RC was strengthened with a single layer of AFRP rods in cross pattern.

The order of specimen ID was chosen to represent specimen number, type of strengthening material, number of layers of strengthening material around the column, shape of strengthening material, and strengthening pattern. For instance, the ID 4-SL-2RC represents specimen #4, strengthened by sisal (SL) rods (R) applied around the column in 2 layers distributed in cross (C) pattern. To distinguish CFRP rods from manually manufactured CFRP rods using CFRP sheets, the letter “R” in specimen 6 and 7 was replaced with the letter “S”. Letters “CP”, “SL”, and “AP” correspond to the presence of CFRP, sisal, and AFRP rods. 

### 2.2. Specimen Details

**Figure 1:**
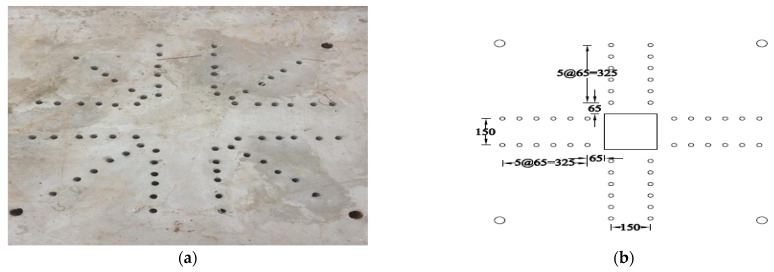
(**a**) Specimen 5-SL-2RS before the installation of sisal rods; (**b**) Position and spacing of drilled holes.

**Figure 2:**
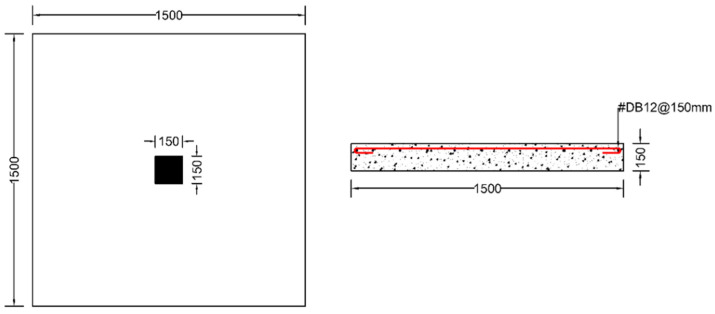
Typical specimen geometry.

Each specimen was cast into a size of 1500×1500 mm. Depth of the specimen was kept as 150 mm. On the tension side, each specimen was reinforced with 12 mm steel bars placed at 150 mm center to center in orthogonal directions. Holes of the size 18–22 mm were drilled into all specimens except specimen #1 to accommodate post-installed shear reinforcement, as shown in Figure 1a. Each layer of holes comprised 6 holes placed 65 mm at the center. The first hole was drilled at a distance of 65 mm from column’s face, as shown in Figure 1b. For specimens strengthened with 2 layers of post-installed shear rods, the distance between the 2 layers was kept at 150 mm. This configuration of holes was kept constant among all specimens. Figure 2 shows typical specimen geometry and cross-sectional details.

### 2.3. Material Properties

**Figure 3:**
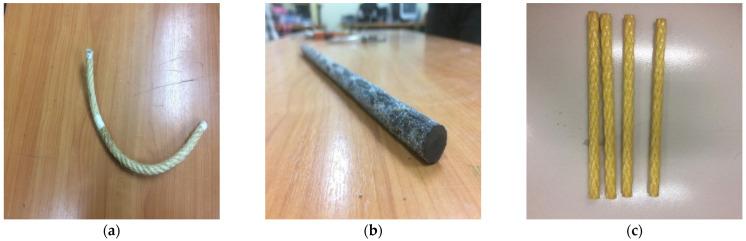
(**a**) Sisal FRP rod (**b**) CFRP rod and (**c**) AFRP rod.

All specimens were constructed from a single batch of ready-mix concrete. The 28-day cylindrical compressive strength of concrete was found to be 31 MPa. The yield and ultimate strengths of longitudinal steel bars were 490 and 460 MPa, respectively. The tensile strengths of CFRP rods, AFRP rods, CFRP sheet, and sisal were 1100, 500, 3500, and 110 MPa, respectively. Sisal, carbon, and aramid FRP rods are shown in Figure 3a–c, respectively.

### 2.4. Specimen Preparation

**Figure 4:**
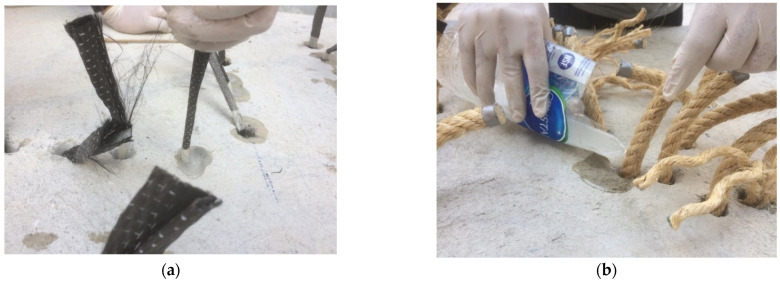
Strengthening of flat slabs, (**a**) CFRP and (**b**) SFRP.

**Figure 5:**
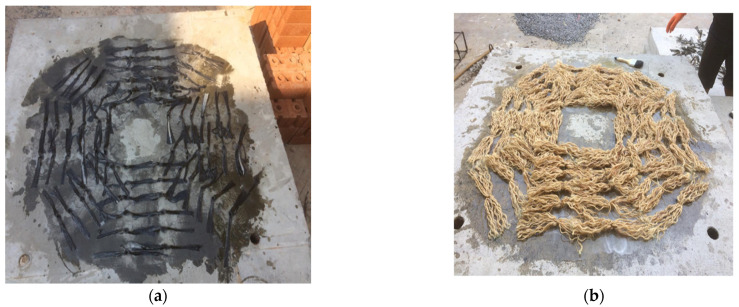
Post-installed (**a**) CFRP sheet rods and (**b**) Sisal rods.

Deformed steel bars (i.e., DB16) were used as the main flexural reinforcement and laid in orthogonal directions. Bars were cut to a length of 1350 mm and laid atop of steel tubes. Steel tubes served a bifold purpose, i.e., to provide a 25 mm bottom concrete cover in addition to serving as holes for the anchorage. Concrete was poured and vibrated simultaneously using a mechanical vibrator. Specimens were cured for 28 days at room temperature. Strengthening of each specimen involved (1) drilling holes at specified locations, (2) inserting the corresponding reinforcement rods in holes, and (3) ejecting liquid epoxy to bond reinforcement with circumferential concrete and seal the holes. Specimens 6 and 7 were strengthened with CFRP rods fabricated using CFRP sheets. For this, CFRP sheets were cut to a length and width of 450 and 60 mm, respectively. CFRP sheets were then folded to the diameter of the drilled holes in slabs. Resin was then applied to CFRP sheet folds at a distance of 150 mm from each end. The middle 150 mm of the CFRP sheet folds was passed through the holes, followed by epoxy injection. Finally, CFRP sheets were unfolded on either side of the slab, as shown in Figure 4a. A similar procedure was adopted to prepare the sisal rods, as shown in Figure 4b. Typical installation of FRP rods is shown in Figure 5.

### 2.5. Load Setup and Instrumentation

**Figure 6:**
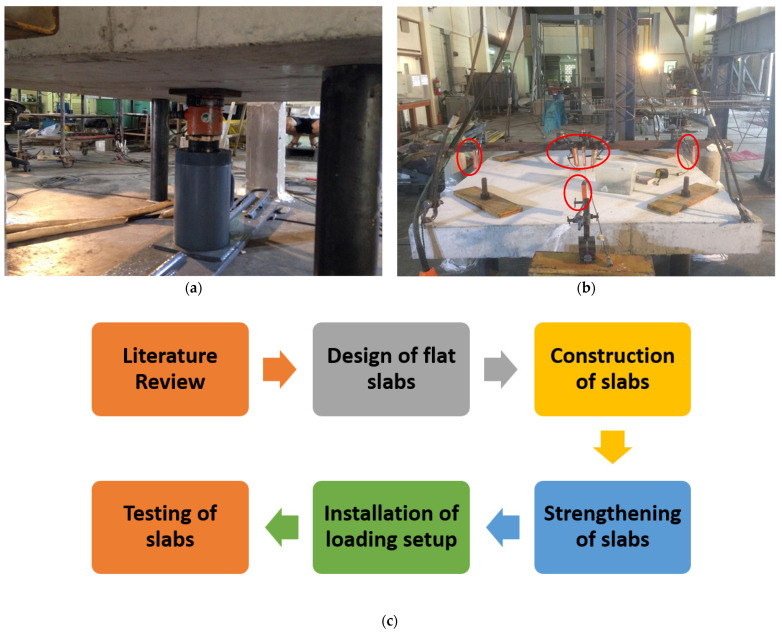
(**a**) Loading configuration (**b**) Locations of LVDTs, (**c**) experimental flow chart.

The loading setup was designed so that the boundary conditions were provided at the corners with steel plates to obtain uniformed and circular boundary conditions for the column. Although there are no direct standards for this kind of loading setup, this method was extensively used in previous studies to investigate the punching shear of flat slabs [23,24]. In this, each specimen was tested in an upside-down position. To simulate the concentric column’s point load, load was applied from the bottom using a hydraulic jack, as shown in Figure 6a. The applied load was concentrated on a local area of 150×150 mm to imitate column’s load. A load cell was coupled to the hydraulic jack to measure the intensity of applied load. A steel section was passed atop of each specimen to facilitate the mounting of Linear Variable Differential Transducers LVDTs. As shown in Figure 6b, 3 LVDTs were fixed at the center of each specimen to measure center deflection. Further, 1 LVDT was attached at the center of each side of the perimeter of each specimen, as encircled in Figure 6b. A simple experimental flow chart is shown in Figure 6c.

## 3. Experimental Results

### 3.1. Crack Patterns and Failure Modes

**Figure 7:**
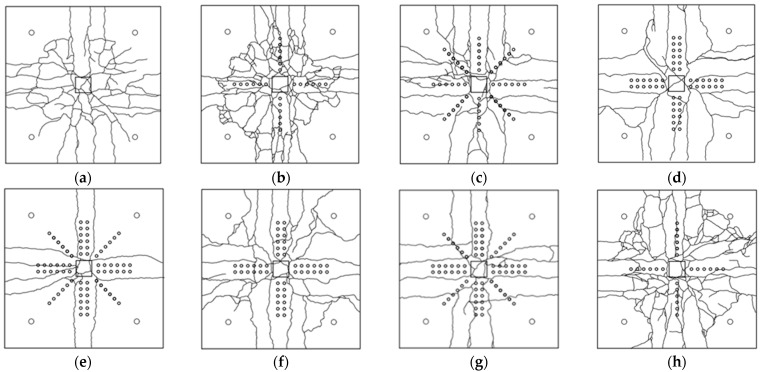
Schematic view of crack patterns at ultimate failure of (**a**) 1-CTRL (**b**) 2-CP-1RC; (**c**) 3-CP-1RS (**d**) 4-SL-2RC (**e**) 5-SL-2RS (**f**) 6-CP-2SC (**g**) 7-CP-2SS (**h**) 8-AP-1RC.

Figure 7 presents a schematic view of the observed crack patterns in all specimens. Loads corresponding to the onset of cracks are listed in Table 2. It can be seen the cracking load for each specimen was approximately 100 kN. Cracks initiated at the center of each specimen and progressed towards edges as the load increased. The control specimen 1-CTRL exhibited the formation of punching cracks at an offset from the central column. Near its ultimate load, the slab was punched down, leading to the failure of the control specimen. Figure 7a shows the ultimate failure of the control specimen. Black thick line corresponds to the formation of the punching cone around the central loaded area. Ultimate failure modes of the remaining specimens are also presented in Figure 7b–h.

Figure 7b indicates the concentration of the damaged zone in the vicinity of the column. A single line of CFRP in cross-pattern was unable to effectively improve the shear capacity relative to the control specimen. In addition, extensive concrete crushing was observed in the compression zone. In the beginning, this crushing was localized within the high-shear-demand zone. Concrete crushing penetrated towards the edges near the ultimate load. Major cracks were observed, initiating diagonally from the corners of the column. The presence of shear reinforcement along the diagonals (Figure 7c) inhibited the formation of these diagonal cracks. Another specimen with single line of shear reinforcement was 8-AP-1RC. Its crack patterns at ultimate failure are shown in Figure 7h. This specimen also experienced extensive damage and a number of cracks were observed at its failure. Nonetheless, the presence of a single line of shear reinforcement inhibited the occurrence of punching failure, as observed in the control specimen.

**Figure 8:**
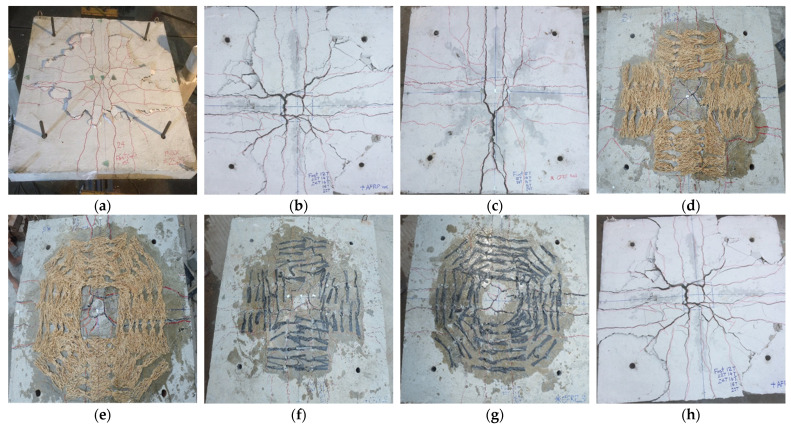
Ultimate failure of (**a**) 1-CTRL (**b**) 2-CP-1RC (**c**) 3-CP-1RS (**d**) 4-SL-2RC (**e**) 5-SL-2RS (**f**) 6-CP-2SC (**g**) 7-CP-2SS (**h**) 8-AP-1RC.

Doubling the shear reinforcement (from a single to double line) improved the behavior of specimens. Figure 7d–g show crack patterns at the ultimate failure of such specimens. These specimens demonstrated a number of flexural cracks running along the lines of shear reinforcement. This improved behavior manifested in the lower number of cracks. Regardless of the number and type of shear reinforcement, crushing of the concrete was observed, initiating from specimen’s center and penetrating towards its edges along shear reinforcement lines as the load increased. The images are shown in Figure 8. The results and crack patterns are consistent with previous studies [23,24].

### 3.2. Load-Deformation Response

**Figure 9:**
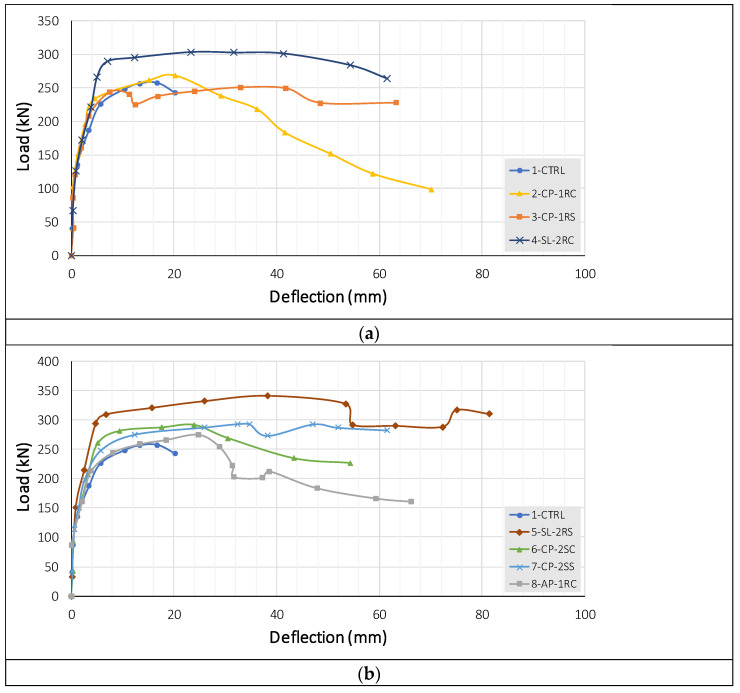

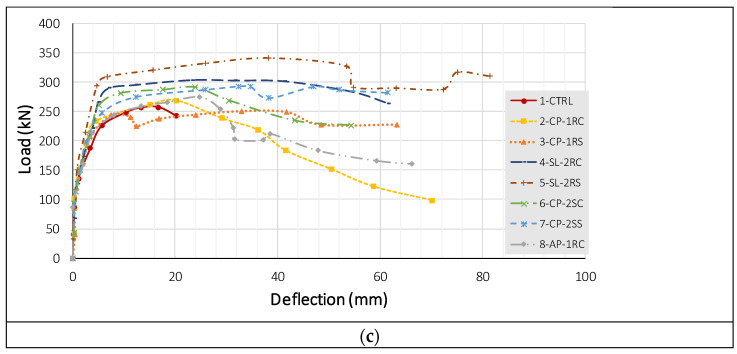
Load-deflection response of all specimens (**a**) comparison between from one to four slabs, (**b**) comparison between one and from five to eight slabs, (**c**) comparison of all slabs.

Experimentally measured load–deflection curves of all slab specimens are presented in Figure 9. Table 2 summarizes important parameters for each curve in terms of cracking load, ultimate laod, and deflection corresponding to ultimate load. Table 2 also presents the gain in ultimate load and deflection as a result of post-installed shear reinforcement. Maximum load sustained by the control specimen 1-CTRL was 245 kN. However, it abruptly dropped its post-peak resistance due to the brittle punching failure.

It is evident from Table 2 that the inclusion of shear reinforcement substantially enhanced the maximum load and failure. An important observation is the deflection at failure loads of strengthened specimens. A significant gap between the deflections at the maximum and ultimate loads of strengthened specimens reflects the ductile behavior imparted by post-installed shear reinforcement. For instance, the gap between deflections at maximum and ultimate loads for the control specimen was approximately 4 mm. This gap increased to approximately 30 mm for some of the strengthened specimens.

In terms of maximum sustained load, single-line shear reinforcement (either cross or star) could only increase upto 6%. This was achieved by the single-line AFRP cross-patterned rods. However, maximum load was increased by up to 31% by the double-line sisal reinforcement applied in the star pattern. The 2nd highest increase in maximum load was up to 17%, which was also triggered by the double-line sisal reinforcement in the cross pattern. Increases in the maximum load by double line cross and star CFRP reinforcement were 12 and 13%, respectively. Nonetheless, it is evident from Figure 9 that double-line shear reinforcement, irrespective of its type, considerably improved the post-peak response, resulting in a ductile behavior. The results in terms of load versus deflection responses are in consistent with previous studies [23,24].

## 4. Analytical Investigation

**Figure 10:**
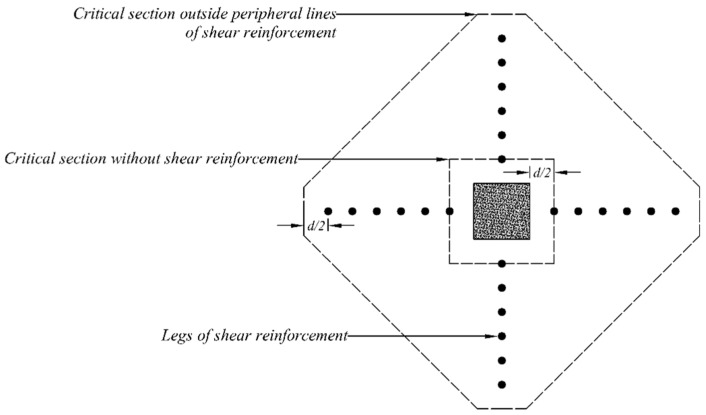
Definition of critical shear sections in two-way flat slabs (single-cross pattern).

**Figure 11:**
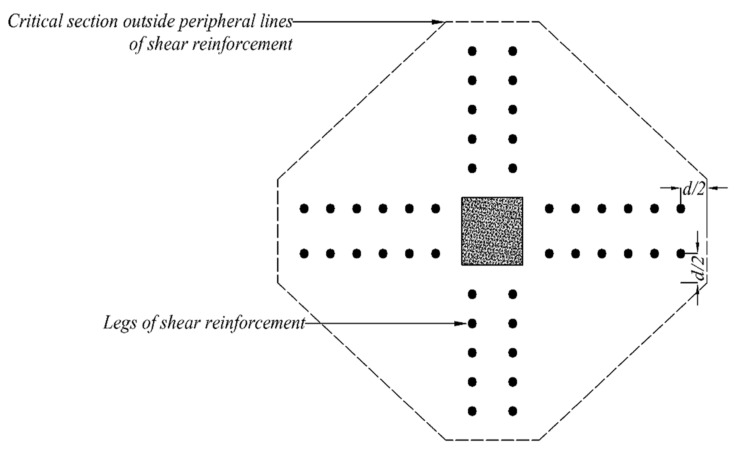
Definition of critical shear sections outside shear-strengthened zone (double-cross pattern).

**Figure 12:**
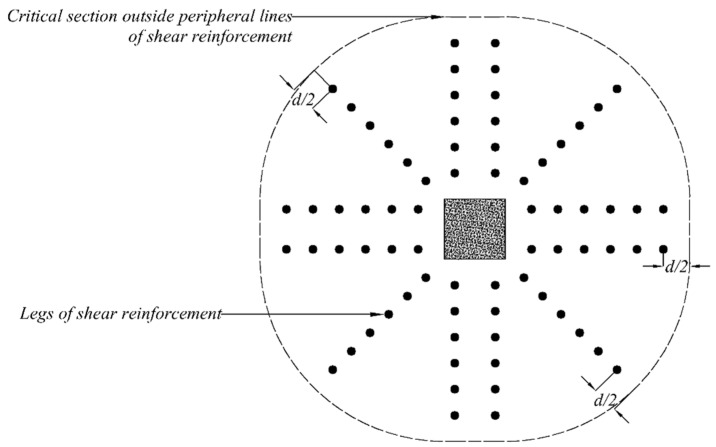
Definition of critical shear sections outside shear-strengthened zone (star cross pattern).

An effort was made to predict the shear capacities of flat slabs with shear reinforcement following the recommendations of ACI 318-19 [47]. The code recommends the presence of a critical shear perimeter at 0.5 times the distance of the effective slab depth from the column’s face. Furthermore, the effect of shear reinforcement on the shear capacity of the slab around columns is also considered. In this case, it is recommended to locate critical perimeter at 0.5 times the distance of the effective depth of slab from the outermost shear stud/rod. Both the cases are graphically represented in Figure 10. A similar procedure was adopted in this study to estimate shear perimeter outside the shear-strengthened zones of star and double cross patterns, as shown in Figure 11 and Figure 12, respectively.

### 4.1. Punching Shear Failure within Shear-Strengthened Zone

Both the concrete and shear reinforcement contribute to shear capacity within the shear-strengthened zone. ACI 318-19 suggests using Equations (1) and (2) to calculate the contribution of concrete and shear reinforcement to the total shear strength of slabs, respectively.

(1) $$V_{c}=min\left[\left(0.33\;\sqrt{{f^{\prime }}_{c}}\right),\;\left(0.083\left(\frac{\alpha_{s}d}{b_{0}}+2\right)\sqrt{{f^{\prime }}_{c}}\right),\;\left(0.167\left(1+\frac{2}{\beta_{c}}\right)\sqrt{{f^{\prime }}_{c}}\right)\right]$$

where:

$V_{c}$ = shear strength contribution due to concrete (MPa);
${f^{\prime }}_{c}$ = concrete cylinder compressive strength (MPa);
d = effective slab thickness for shear (mm);
$b_{o}$ = perimeter of shear critical section 0.5d from loading area periphery (mm);
$\alpha_{s}$ = factor according to the type of connection; it is 40 for internal columns, 30 for external columns, and 20 for corner columns;
$\beta_{c}$ = ratio of the long side to the short side of loading area periphery.

(2) $$V_{s}=\frac{A_{v}f_{yt}}{b_{0}s}$$

where:

$V_{s}$ = shear strength contribution due to reinforcement (MPa);
$A_{v}$ = sum of the area of all shear reinforcement in one peripheral line;
$f_{yt}$ = yield strength of shear reinforcement;
s = spacing between consecutive peripheral lines of shear reinforcement parallel to loading area periphery.

ACI 318-19 states that the shear resistance provided by the concrete in the presence of shear reinforcement must be reduced from $0.33\;\sqrt{{f^{\prime }}_{c}}$ to $0.25\;\sqrt{{f^{\prime }}_{c}}$. Total shear resistance $\left(V_{t}\right)$ of the slab is obtained by the summation of Equations (1) and (2) with the upper limit specified in Equation (3) to account for concrete diagonal crushing.

(3) $$\;\;V_{t}=V_{c}+V_{s}\leq 0.67\;\sqrt{{f^{\prime }}_{c}}\;\mathrm{MPa}$$

Inherently, composite materials remain elastic till their fracture point. To account for the contribution of composite/natural shear reinforcements in this study, Equation (2) was modified to replace $A_{v}$ and $f_{yt}$ with their corresponding areas and fracture strengths. Previous work [24] carried out on the shear strengthening of flat-plate slabs with Glass Fiber-Reinforced Polymer (GFRP) rods suggested that CFRP and GFRP rods attained only about 25% of their ultimate strain at slab failure. The same value was adopted in this study by limiting the fracture strengths of shear reinforcements by up to 25% of their values.

### 4.2. Punching Shear Failure outside Shear Strengthened Zone

ACI 318-19 predicts that the shear strength offered by the slab outside the shear-strengthened zone would consist only of the concrete contribution, and is given in Equation (4).

(4) $$\;V_{c}=0.167\sqrt{{f^{\prime }}_{c}}\;\mathrm{MPa}$$

Table 3 presents a comparison between experimental and analytical shear capacities. It should be mentioned that $V_{c\left(in\right)}$ stands for the punching capacity of specimens within the shear-strengthened zone and is calculated from Equation (3). It can be seen that ACI predictions of shear strength outside the shear-strengthened zone were from 10 to 30% higher than experimental maximum loads, except for Sisal-strengthened specimens, i.e., specimens 4-SL-2RC and 5-SL-2RS. The ratio $V_{c\left(in\right)}/P_{exp}$ of CFRP- and AFRP-strengthened specimens ranged from 1.56 to 2.02, indicating that sufficient shear strength was mobilized by these composites within the vicinity of columns. Furthermore, their corresponding $V_{c,out}/P_{exp}$ were relatively closer to 1, indicating that shear failure zone was successfully shifted outside their shear-strengthened zones. In the past, Yooprasertchai et al., 2021, also modified American Concrete Institute (ACI) recommendations to predict the contributions that GFRP rods make to the punching shear-strengthening of flat slabs [23]. However, in this study, this result is in good agreement with their corresponding failure mechanisms. Except for Sisal, analytical predictions of all the strengthened specimens were higher than their corresponding maximum loads. It can be concluded that ACI 318-19 overestimates the actual shear strengths of the strengthened specimens and the factor of 0.167 must be adjusted to a lower value. A 25% limit was imposed on the participation of shear reinforcements (irrespective of their type) in the ultimate strain, based on the experimental work of [24]. This value clearly induces erroneous analytical predictions of experimental results. Effective strains in various rods at the onset of shear failure must be quantified in future studies to furnish suitable reduction factors in Equation (2). Therefore, further studies are required to confirm the validity of these results and propose equations to estimate the shear strengths of flat slabs strengthened with composite or natural post-installed reinforcements.

## 5. Conclusions

This study carried out a comparative experimental program to examine the effects of post-installed CFRP, AFRP, and sisal rods in flat slabs. From the experimental results of eight specimens presented in this study, the following important conclusions can be drawn.

1. Control specimen suffered a brittle punching failure owing to its sub-standard detailing. On the contrary, all the strengthened specimens, irrespective of the type of shear reinforcement, successfully mitigated punching failure.
2. Another important observation is the deflection of strengthened specimens at failure loads. The significant gap between the deflections at maximum and ultimate loads of strengthened specimens reflects the ductile behavior imparted by post-installed shear reinforcement. For instance, the gap between deflections at maximum and ultimate loads for the control specimen was approximately 4 mm. This gap increased to approximately 30 mm for some of the strengthened specimens.
3. In terms of the maximum sustained load, the single-line pattern (cross or star) could only increase the resistance over that of the control specimen by 6%. A substantial difference in the maximum sustained load was observed when the amount of post-installed reinforcement was increased from single- to double-line, regardless to its type.
4. For equivalent areas of CFRP, AFRP, and sisal reinforcement, sisal reinforcement resulted in the maximum improved performance. This, too, was achieved by inheriting the lowest ultimate strength of sisal (i.e., 110 MPa).
5. Analytical assessment was carried out, as per the recommendations of ACI-318-19. For the control specimen, ACI equation resulted in a close approximation. For flat slabs strengthened with shear reinforcement, ACI explicitly provides equations to estimate shear strength by incorporating an enlarged critical perimeter. ACI equation overestimated the punching shear strengths of CFRP- and AFRP-strengthened specimens by up to 30%. On the contrary, the shear strengths of sisal strengthened slabs were underestimated. Therefore, further experiments should be conducted to validate these experimental results and propose equations for the shear strength of flat slabs strengthened with composite or natural post-installed reinforcements by explicitly accounting for their type and ultimate strengths.
6. This study has shown that the use of CFRP, AFRP and sisal FRP rods is very useful and affordable solution for strength enhancement. Therefore, the outcome of this study is very useful, and the proposed methods can be further used for the economical and safe strengthening of flat slabs.

## Figures and Tables

**Table 1:** Test matrix.

Specimen ID	Strengthening Material	Strengthening Pattern	Number of Layers	Schematic View
1-CTRL	-	-	-	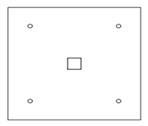
2-CP-1RC	Carbon FRP rod	Cross	1	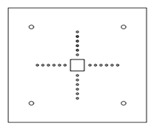
3-CP-1RS	Carbon FRP rod	Star	1	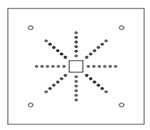
4-SL-2RC	Sisal	Cross	2	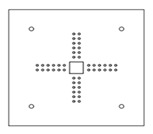
5-SL-2RS	Sisal	Star	2	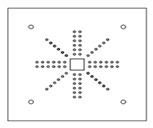
6-CP-2SC	Carbon FRP sheet rod	Cross	2	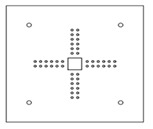
7-CP-2SS	Carbon FRP sheet rod	Star	2	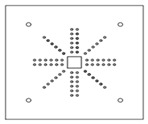
8-AP-1RC	Aramid FRP	Cross	1	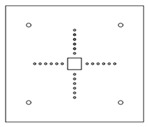

**Table 2:** Experimental load-deflection values at maximum and ultimate loads.

Specimen ID	First Crack		Maximum		Failure		Gain at Maximum		Failure Mode
	Load (kN)	Deflection (mm)	Load (kN)	Deflection (mm.)	Load (kN)	Deflection (mm.)	Load (%)	Deflection (%)	
1-Ctrl	100	0.46	258	15.1	245	19.5	-	-	Shear
2-CP-1RC	120	0.69	267	18.82	200	40	3	24	Flexure
3-CP-1RS	100	0.11	250	33.95	240	68	−3	124	Flexure
4-SL-2RC	140	1.21	304	29.77	280	60	17	97	Flexure
5-SL-2RS	120	0.32	339	36.55	310	78	31	142	Flexure
6-CP-2SC	120	0.61	291	23.46	240	54	12	55	Flexure
7-CP-2SS	120	0.79	293	46.79	280	60	13	209	Flexure
8-AP-1RC	110	0.32	276	23.37	240	28	6	54	Flexure

**Table 3:** Comparison between experimental results and analytical predictions.

ID	$b_{o\left(out\right)}\;(\mathrm{mm})$	$V_{c\left(in\right)}\;\;(\mathrm{kN})$	$V_{c\left(out\right)}\;\;(\mathrm{kN})$	$P_{exp}\;\;(\mathrm{kN})$	$V_{c\left(in\right)}/P_{exp}$	$V_{c\left(out\right)}/P_{exp}$
1-Ctrl	-	248	0	258	0.96	-
2-CP-1RC	3152	504	381	267	1.89	1.3
3-CP-1RS	3472	504	420	250	2.02	1.2
4-SL-2RC	3092	294	374	304	0.97	0.8
5-SL-2RS	3460	347	418	339	1.02	0.8
6-CP-2SC	3092	504	374	291	1.73	1.3
7-CP-2SS	3460	504	418	293	1.72	1.2
8-AP-1RC	3152	429	381	276	1.56	1.1

## Data Availability

The data presented in this study are available on request from the corresponding author.
